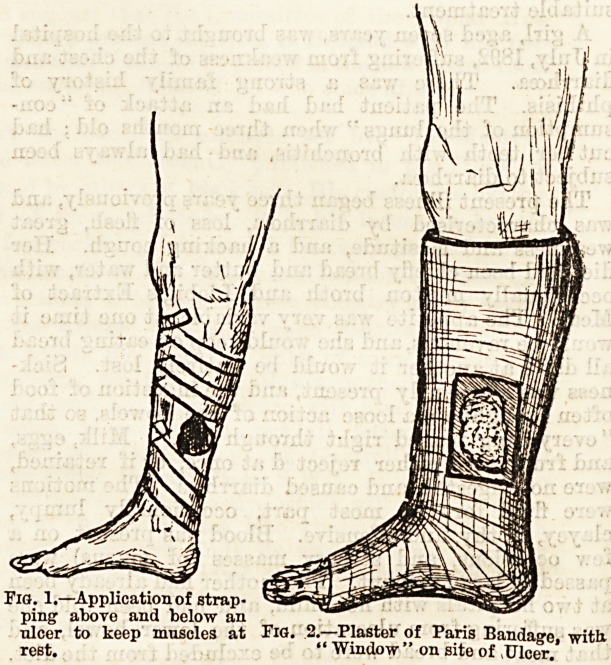# The Treatment of Some Forms of Chronic Ulcer

**Published:** 1893-09-02

**Authors:** 


### LONDON HOSPITAL.

### THE TREATMENT OF SOME FORMS OF CHRONIC ULCER.

The fundamental idea that underlies all treatment of chronic ulcer is the endeavour to obtain as quickly and efficiently as may be a return of the tissues of the affected part to their healthy condition of growth, regeneration and change. And in bringing this about we have to beware of letting our attention dwell too closely on the local change, for behind this local lesion there must be some more general cause preventing the tissues at the affected spot living their life of healthy cell growth and organisation, filling up as healthily as may be the limited destruction of the body covering called an ulcer. Growth and change are always taking place about an ulcer, but because the products of growth are abnormal or deficient—abnormal in producing pus, deficient in that growth stops at granulation tissue instead of developing into connective tissue, and that the skin around the sore grows badly or not at all, failing to cover the part with a new protective coating. We then see that in every case our attention has to be directed to three main factors influencing the healing or persistence of an ulcer. These are the state of health of the individual, the state of rest of the affected part, and the local condition of the ulcer and tissues round it; attention to one of these points is useless while the others are neglected.

It has long been recognised here that attention must be paid to the general health, and this rule still holds good in cases of chronic ulcer where no constitutional disease requiring specific treatment is present. While the blood is anæmic and the digestive functions below par, it is impossible to expect that tissues whose vitality is already impaired can take on healthy local action and repair. So, in cases of chronic ulcer we correct what digestive disturbance there may be and regulate the workings of the intestinal tract, while supervising carefully the feeding and hygiene of the body. Iron is given if the state of the blood seems to demand it, bearing in mind that iron is useless while the intestinal tract is inactive.

In some cases of chronic ulcer about the extremities, very small doses of tincture of opium once or twice a day have been used for many years, empirically at first, then with the idea that they had some effect in improving the peripheral circulation, empirically or scientifically, often with decided benefit. With the exception of ulcers of specific origin, tubercular ulcers seem most to require definite constitutional treatment, and for these the syrup of iodide of iron is a favourite preparation, added to cod liver oil or malt extract.

Another point that has to be considered is the state of passive congestion of the part; this more especially in those chronic ulcers about the legs associated with varicose veins, and often beginning as a little eczematous patch caused by the congestion, and irritated into an ulcer by rubbing or scratching. In these cases, with the parts overloaded with venous blood, repair is slow or absent, and healthy growth cannot take place till better circulation is established. Rest in bed is best when practicable; if not, either some operation on the veins, if they are large enough to cause danger and discomfort, obliterating in some way their lumen at several points so as to break the weight of the column of blood. In slighter cases an elastic stocking or Martin's perforated rubber bandages are used, the latter being rolled round the limb over ulcer and all before getting up in the morning, without stretching the bandage; when the leg fills with blood after rising the bandage will be found tight enough. The ulcer may be dressed with any simple wet dressing. Boracic lint answers well, ointments being avoided as they destroy the rubber of the bandage.

In a fair proportion of cases the rubber bandages are not tolerated, as they confine the perspiration, causing considerable local irritation, and if there is already any eczema tending to increase it. In such cases an elastic stocking may be tolerated when a bandage cannot be worn, and either one or the other should be advised as prophylactic treatment, when with varicose veins there is any tendency to eczematous ulceration.

The pressure of the Martin's bandage is found to conduce directly in bringing about a healthy condition of the ulcer in cases where the granulations are exuberant, flabby, and watery. Another form of dressing often used in these cases with excellent results is the application of Unna's dressing to the limb. Unna's paste which is used for this purpose is a mixture of zinc oxide 5 parts, gelatine 6 parts, glycerine 8 parts, water 6 parts, melted together in a water bath, and well stirred. The ulcer is covered with a piece of boracic lint, and the limb strapped from the toes up, with strips of gauze the size and shape of the pieces of adhesive plaister used for strapping a limb. As each piece is applied it is painted over with the paste, heated over the fire or in hot water till it is in a fluid condition; the whole limb is covered in this way, over ulcer and all; a second layer being applied in a similar manner, it sets fairly quickly, and is left on for a week, when it can be easily cut off with a pair of scissors. The granulations on the floor of the ulcer will be found smaller, firmer, and healthier, and the skin from the margin will have begun to grow in over the granulating surface. Some astringent dressing, such as nitrate of silver lotion (two grs. to ounce), or ointment of the red oxide of mercury, is applied for a time, alternating if there is much discharge, with boracic dressings, till the granulations are small and firm, when, if the ulcer is of any size, a few skin grafts will hasten the healing.

We now turn to a very large class of cases, met with chiefly among the labouring population, where a large callous ulcer, usually of the leg, eating deeply into the tissues, has persisted for many years, often in a patient who has to continue at work

Fig. 1.—Application of strapping above and below an ulcer to keep muscles at rest.

Fig. 2.—Plaster of Paris Bandage, with "Window" on site of Ulcer.

as long as possible, and to whom the recommendation to rest in bed for eight or ten weeks would be little better than a farce. As long as the muscles of the legs are constantly in action it is useless to hope for any great amount of healing to take place, the granulations on the floor of the ulcer being so constantly disturbed. First, rest of the part must be obtained as much as possible; this can be ensured to a considerable extent by means of strapping the limb above and below the ulcer (fig. 1), a method commonly adopted. Better still is the application of plaster of paris to the limb above and below the ulcer, fixing the muscles, and keeping them at rest. This is applied as follows: A piece of stout cardboard cut to the size of the ulcer is placed over it, a piece of lint with zinc ointment having been first placed over its floor, the whole limb bandaged with a domette bandage; having carefully noted the site of the ulcer, the limb from the toes up is now bandaged with muslin bandages impregnated with plaster of paris and wetted, till they are four or six layers deep over the whole limb. When the plaster has set, a "window" is cut out over the site of the ulcer equal in extent to its area, the cardboard protecting the skin from injury while this is being done (fig. 2). The margins of the window are painted either with a solution of gutterpercha in chloroform, or melted solid paraffin and beeswax (equal parts), the object being to keep discharges from the ulcer getting down the limb inside the plaster. Dressings can now be applied to the ulcer, though the muscles are kept in a condition of absolute rest; a piece of stout guttapercha splinting is cut to just fit into the window, and is kept in place over the dressings with a turn or so of bandage. This keeps up a necessary and stimulating pressure on the granulations, and prevents them growing too exuberantly, obviating at the same time any tendency to a local œdema of the unsupported area, either a watery astringent dressing, nitrate of silver, sulphate of zinc or copper, or some stimulating ointment, such as red oxide of mercury, or zinc with benzoin being applied to the ulcer. With the rest ensured by the plaster, and the stimulating treatment, the granulation tissue can be made to take a healthier action in a great number of cases, and healing eventually brought about.

Sometimes, when after a trial of various applications an ulcer does not heal, either from its size, position, or callousness, grafting is performed, either with small skin grafts or larger layers of epidermis by Thiersch's methods, both of which give excellent results.

Another form of chronic ulcer is that associated with tubercle, and often proving excessively intractable, either in not healing or breaking down again almost immediately. If possible, sea air, especially Margate, is of inestimable benefit in promoting the healing of these ulcers, and, whenever possible, the patient is sent to the seaside. Scraping off the caseous floor of the ulcer with Volkman's spoons, trimming away the livid, overhanging edges of useless, half-dead skin, with the application of iodoform in powder or as ointment, are the means most relied upon to bring about a healthier condition, any basis for the ulcer in a neighbouring suppurating and caseous gland being looked for and removed; improved nutrition by good food and better personal hygiene being even more important than local treatment.

## Figures and Tables

**Fig. 1. Fig. 2. f1:**